# Data of the freezing curves of tuna blocks with or without the weak oscillating magnetic fields

**DOI:** 10.1016/j.dib.2020.105852

**Published:** 2020-06-14

**Authors:** Kana Okuda, Aiko Kawauchi, Kentaro Yomogida

**Affiliations:** a Mukogawa Women's University, Ikebiraki-cho 6-46, 663-8558 Nishinomiya, Japan; b Abi Inc., Ohtakanomori-higashi 1-12-1, 270-0138 Nagareyama, Japan

**Keywords:** Freezing curves, Tuna blocks, Air blast freezing, Oscillating magnetic field, Freezing time, Crystallization time;Freezing rate

## Abstract

Although freezing is the most popular method for long-term food preservation, the formation of ice crystals during the process often leads to degradation of the product quality. Recently, we demonstrated that the presence of oscillating magnetic fields (OMFs) can hinder ice crystallization (10.1016/j.cryobiol.2020.05.005, [1]). In this data that we investigated the effects of OMFs on freezing tuna blocks using the Cell Alive System^Ⓡ^ (CAS^Ⓡ^) (ABI Co. Ltd., Chiba, Japan) developed as a rapid freezer unit supplemented with an OMF generator. The center temperature of tuna blocks was monitored during air blast freezing (ABF) or ABF combined with CAS^Ⓡ^ (ABF-CAS). The time taken to acquire the freezing temperature (-20 °C) was significantly (*p* < 0.05) shortened with ABF-CAS compared to ABF. The time taken for ice crystal formation (crystallization time) was slightly shorter in case of the ABF-CAS system relative to ABF (*p* = 0.08497).

Specifications table

**Table:** 

Subject	Food Science
Specific subject area	Food preservation and freezing/thawing processes
Type of data	Tables Figure
How data were acquired	Real time monitoring of temperature and estimation of freezing time
Data format	Raw Analyzed
Parameters for data collection	The data for the freezing curves were collected at two conditions. The first condition corresponds to freezing by the commonly used air blast method, in which the freezer was precooled to −30 °C and the air temperature was set to −50 °C (rapid freezing mode) with 50% airflow (approximate rate of 0.5 m/s). The second condition employed the same air blast method in the presence of oscillating magnetic fields.
Description of data collection	We monitored the temperature at the center of the tuna blocks during the freezing process in real time using a type K thermocouple connected to a temperature recorder. The temperature inside the freezer was also monitored in real time by a similar method. The obtained data were exported to Microsoft Excel (Office 365), followed by analysis and plotting according to some existing definitions of freezing time.
Data source location	Mukogawa Women's University Nishinomiya-shi, Hyōgo-ken, Japan Geographic coordinates: 34.716^°^ N (latitude) and 135.37^°^ E (longitude)
Data accessibility	All relevant data is included in the article.
Related research article	Authors: Kana Okuda, Aiko Kawauchi, Kentaro Yomogida Title: Quality improvement of frozen mackerel (*Scomber japonicus*) muscle tissue frozen by the rapid freezer with the weak oscillating magnetic field Journal: *Cryobiology* DOI: 10.1016/j.cryobiol.2020.05.005

## Value of the data

• This research represents the first report on the shortening of freezing time (time taken to acquire the freezing temperature) during animal tissue freezing by oscillating magnetic fields using real fish tissues with complex internal structures.
• These data provide enhanced understanding of factors contributing to structural damages in frozen food.
• These data hint that the suppression of latent heat during tissue freezing is a prominent factor affecting tissue damages induced by ice crystallization.
• These observations provide useful insights into safe tissue cryopreservation required in the field of regenerative medicine.

## 1 Data description

Details of tuna (*Thunnus albacares*) blocks used to data the freezing process are summarized in Table 1.

**Table 1:** Details of each tuna block.

Freezing conditions	Weight (g)	Thickness (mm)
ABF-CAS 1	45.68	16.71
ABF-CAS 2	46.06	13.74
ABF-CAS 3	43.16	12.46
ABF-CAS 4	54.84	17.25
ABF-CAS 5	55.40	14.17
ABF 1	47.49	12.48
ABF 2	43.32	13.20
ABF 3	53.65	15.67
ABF 4	48.18	16.98
Ave.	48.64	14.74
SD	4.80	1.94

**Fig. 1:**
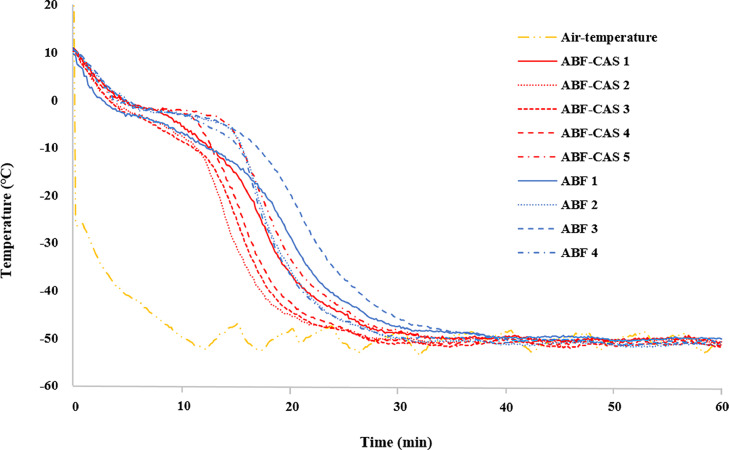
The time-dependent freezing curves for tuna blocks frozen by air blast with or without CAS were recorded at the center of the frozen sample (*n* = 4–5 in each test).

The time-dependent freezing curves for tuna blocks frozen by air blast with or without CAS (ABF-CAS and ABF, respectively) were recorded at the center of the frozen sample (Fig. 1).

Data obtained from the curves were evaluated for freezing characteristics according to the respective definitions (detailed in Table 2).

**Table 2:** Estimation of freezing time.

	Freezing time(min)	The time take to acquirethe freezing temperature (−20 °C)(min)	Crystallizaiton time(min)	Freezing rate( °C/min)
ABF	**35.6** **±** **5.6^a^**	**19.3** **±** **1.7^a^**	**8.8** **±** **1.4^a^**	**1.7** **±** **0.6^a^**
ABF-CAS	**31.1** **±** **3.2^a^**	**17.1** **±** **1.6^b^**	**6.53** **±** **2.0^a^**	**2.5** **±** **1.4^a^**

Data are expressed as mean ± SD. Different letters for mean values within a column represent significant different (*p* < 0.05).

The time to freezing temperature (−20 °C) in ABF-CAS was significantly (*p* < 0.05) shorter than that in ABF. The time taken for ice crystal formation was also slightly shorter for CAS-ABF compared to ABF (*p* = 0.08497). The freezing time (time required to acquire the set temperature of −50 °C; *p* = 0.10768) and freezing rate (*p* = 0.29294) was similar in tuna blocks frozen by CAS-ABF and ABF methods.

## 2 Experimental design, materials, and methods

Freeze-thawed tuna blocks were purchased from a local supermarket in Japan. The tuna samples (each about 50 g in weight, 6 cm in length, 5 cm in diameter, and 15 mm in thickness) were cut rectangular. These samples were selected randomly for testing out the different freezing conditions.

The different experimental batches were frozen with an air blast freezer with and without the CAS^Ⓡ^ system. The freezer used in this data had been designed in consultation with ABI (F201003026, ABI Co., Ltd., Chiba, Japan), with the OMFs and control system supplied and commissioned by ABI. The basic construction of the freezer consisted of a cooling unit, a freezing cabinet, 2 fans, and 2 control panels. The freezing cabinet contained a rack with 10 equidistant rails that would accommodate up to 10 aluminum trays for freezing food, along with the magnetic field generator. Different freezing conditions, namely air temperature (−50 °C), airflow (0/100%), and ‘CAS energy’ (0/700), could be set by the control panel [1].

The freezer was precooled to −30 °C (precooling mode), and the tray with samples was arranged in a single layer in the middle of the cabinet. After the sample was frozen, the air temperature was decreased to −50 °C (rapid freezing mode) with airflow of 50% (corresponding to a flow rate of approximately 0.5 m/s). With CAS^Ⓡ^ freezing, the CAS energy was set at 500, as per the recommendation by ABI. The CAS^Ⓡ^ energy was turned off for samples frozen only with ABF. The OMF strength was measured using a gaussmeter (EMF-828, Fuso Co. Ltd., Tokyo, Japan). The OMF strength with CAS energy could be controlled in the range of 0.1 to 1.0 mT. The OMF strength in the ABF-CAS mode recommended by ABI for raw fish was precisely controlled to ensure a working range of 0.1 to 0.5 mT. The freezing process was considered complete when the temperature at the center of the tuna block reached −50 °C.

The temperature at the center of all samples was monitored in real time during the freezing process using a type K thermocouple (0.32 mm diameter 1 G, NND Co., Ltd., Japan) connected to a temperature recorder (MSR128-V6, M-System Co., Ltd., Japan). An accurate insertion of the thermocouple at the center of all the samples was ensured. In addition, the frozen samples were defrosted and dried after completion of the first freezing step for restoration to the initial state, followed by the second freezing step. The obtained data was output in the CSV format and analyzed for estimating the freezing time.

As samples gradually froze in an inward direction from the surface to the center, it was difficult to determine precisely the total freezing time. The temperature at the center was dissipated outside due to the latent heat associated with crystallization of ice during freezing. Consequently, we defined the “freezing time” as the time taken for the center of the sample to reach the set temperature from the time of initiation of freezing. The “time to freezing temperature” was considered the time taken for the temperature at the center to reach −20 °C from the initial temperature, because commercially available frozen food has a temperature of −18 to −20 °C. In addition, we defined the crystallization time as the time taken for the temperature at the center of the sample blocks to pass through the crystallization temperature zone (−1 to −7 °C) [2]. It has been suggested that the final quality of the frozen sample could be evaluated based on the freezing rate. We calculated the freezing rate from the time taken for the temperature at the center to decrease to −10 °C from −5 °C, as described previously [3], [4], [5].

Experimentally acquired data were processed by means of variation statistics, namely mean and standard deviation (SD). To compare the parametric data in the two groups, a *t*-test was used. Statistical analysis was performed using the Student's *t*-test, and differences between the groups were considered statistically significant when the P value was less than 0.05.

## Declaration of Competing Interest

The authors declare that they have no known competing financial interests or personal relationships which have, or could be perceived to have, influenced the work reported in this article.
